# Values and ethics for implementation fidelity and integrity of public health policy in a multipolar world with conflicting value systems

**DOI:** 10.1093/pubmed/fdaf128

**Published:** 2025-11-18

**Authors:** Joseph Mfutso Bengo, Eva Mfutso Bengo

**Affiliations:** Department of Bioethics, Behaviour and Health Social Sciences, Kamuzu University of Health Sciences, Mahatma Gandhi Road, Blantyre 31225, Malawi; Department of Economics and Law, School of Business and Economic Sciences, Malawi University of Business and Applied Sciences, Chichiri, Blantyre 31225, Malawi

**Keywords:** management and policy, public health, cultural identity

## Abstract

Global/public health policy and security historically relied on alignment of values to the Western value system to achieve public health security, environmental protection, emergencies, and disaster-risk management and public health governance. This alignment was facilitated by globalization. As geopolitical landscapes shift, a clash of values is eroding the dominance of once-uniform systems. This is paving the way for a multipolar world, where diverse value systems increasingly influence public health policies and discourse. Whereas evidence informs policy decisions, values motivate public health implementation integrity and fidelity. Ethical values and law are indispensable pillars of good public health policy, implementation and practice. The African *Ubuntu* ethical value system recognizes that being human is to be interrelated, interdependent and interconnected with all for all. It accommodates diversity in unity through harmonization, reciprocity, solidarity and the common good. It is in line with the One Health concept. This commentary proposes shared ethical values as a tool for implementation integrity and fidelity of global/public health policy. It recommends value- and evidence-based public health decision-making and practice that recognizes the paradigm shift from value alignment based on uniformity to harmonization of values based on unified diversity and moral reasoning.

## The aspiration of harmonized values: a post-war ideal

The mid-20th century, emerging from the devastation of two World Wars, witnessed a concerted effort towards establishing universal norms and values to prevent future global catastrophes.^1^ This era was characterized by a profound belief in the possibility of harmonizing diverse interests to achieve shared goals such as global trade, public health security, environmental protection, and effective emergency and disaster response. The Universal Declaration of Human Rights, adopted in 1948,^2^ stands as a testament to this aspiration, enshrining human dignity as a global common good.

The International Covenant on Economic, Social and Cultural Rights (1966) implicitly demands global cooperation to ensure the right to a standard of living adequate for health and well-being (Article 11) and the highest attainable standard of health (Article 12),^3^ even though it does not explicitly mention international collaboration for emergencies. This foundational commitment to universal values permeated the emerging field of public health. The Alma Ata Declaration of 1978,^4^ for instance, championed primary health care values rooted in equity, social justice, and community participation. Similarly, the WHO International Health Regulations reflect this spirit.^5^ Concurrently, ethical guidelines such as the Nuremberg Code (1947), the Declaration of Helsinki (1964),^6^ the CIOMS International Ethical Guidelines for Health-related Research Involving Humans^7^ (2016), and UNESCO's Universal Declaration on Bioethics and Human Rights (2005), along with various WHO guidelines all represented attempts to standardize, harmonize, and amalgamate diverse ethical principles and values into universally acceptable standards.

This pursuit of global value uniformity was largely influenced and driven by an economic, political and cultural hegemony based on Western value system. This globalization-based world order was seen as serving Western interests at the time.^8^

## The ‘clash of values’: from unipolarity to multipolarity

The optimistic vision of a seamlessly harmonized global value system, however, has faced increasing scrutiny. While globalization initially fostered an amalgamation of cultural values, often leaning heavily on Western liberal democratic ideals following the collapse of the Soviet Union and the emergence of a unipolar world, this dynamic has begun to unravel. Samuel Huntington's influential work, The Clash of Civilizations and the Remaking of the World Order (1996),^9^ highlighted potential conflict stemming from divergent cultural values and civilizational identities.One can reinterpret this ‘clash of civilizations’ as a ‘clash of values,’ where the previously assumed universality of Western values is being challenged. This challenge is also reflected in the intense intellectual discourse on decolonizing global and public health.^10^

The rise of regional and national identities, exemplified by phenomena such as Brexit, and the formation of blocs like the African Union (AU) and BRICS (Brazil, Russia, India, China, South Africa), signify a global shift away from a singular, hegemonic value and economic system.^11^ For decades, particularly after the Cold War, the West, specifically the United States, emerged as the dominant global power, setting trends in politics, finance, culture, technology and public health.^12^ This unipolar world implicitly championed a uniformity of values, where Western standards and practices became de facto global norms.

However, the emergence of a multipolar world, with the growing influence of Brazil, Russia, India, China, South Africa and other nations, has provoked a significant departure from this unipolar paradigm. This shift is demonstrated by increasing criticism of Western hegemony, uniformity, and dominance in global politics and finance, which consequently also affects global/public health. The notion that global/public health practice, economics, and governance should uniformly adhere to Western-derived principles is now being actively resisted.

Paradoxically, the rise of this multipolar world, with its inherent diversity, has also triggered a reaction from some Western quarters, manifesting as a form of anti-globalization. For instance, ‘America First’ policies are a strong manifestation of this nationalistic, conservative, and religious approach that challenges liberal and often secular frameworks.

This anti-globalization sentiment is often characterized by a renewed emphasis on national interests and a questioning of the global frameworks and value alignments that underpinned the previous era of globalization. Within this anti-globalization wave lies a significant clash of values between conservative value systems and liberal value systems, impacting international cooperation and the very fabric of global and public health programs and research.

Hence, there is not only a geographical value divide between countries, but also a value divide within societies.

## Multipolar ethos, ethical uncertainty and the search for unified diversity

Public health professionals will be increasingly confronted with the need to find common ethical principles, value sets and codes of ethical conduct for public health policy and practice, even as cultural identities diverge and societies polarize. This paradigm shift offers an opportunity to integrate diverse cultural perspectives into global public health discourse. However, it also raises ethical uncertainty about whether a common ethical consensus can be reached. The challenge is to find a framework based on moral reasoning and robust arguments, one that avoids the pitfalls of unipolar or multipolar power dynamics. In Ubuntuology,^13^ this process would be called ‘unified and reconciled diversity’. The philosophy of *Ubuntu*,^14^ originating from African ideals, posits that existence itself is relational. As humans we are inherently interdependent, interconnected, and interrelated with all beings and the environment. This relationship includes past, present and future, physical and meta-physical beings and world. This is encapsulated in the saying, ‘We are therefore I am; I am therefore we are.^15^ Hence, Ubuntuology provides a robust philosophical foundation^16^ for the ‘One Health’ concept,^17^^,^^18^ recognizing that spiritual, human, animal, and environmental health^19^ are ontologically interrelated, interdependent and interconnected, sharing a common destiny on a single universe.

This holistic worldview contrasts sharply with individualistic approaches that have often characterized Western public health models. The principle of interdependence of human beings for the common good is reflected in many other cultures, religions and philosophies, including in the principle of solidarity^20^ (also part of Western thought^21^), Christian social teaching,^22^ Islam,^23^ Judaism,^24^ Confucianism.^25^ This shows the possibility of convergence of values for the common good of public health.

## The indispensable role of values for implementation fidelity and implementation integrity

The preceding discussion demonstrated how value clashes can cause conflict and disagreement, affecting public health decisions, collaboration, and implementation. It's crucial to acknowledge that values can be social, personal, cultural, religious, professional, and ethical. While evidence is paramount for optimizing public health decisions, ethical values are equally essential for achieving both public health implementation fidelity and integrity. All forms of behavior, including policy-making and implementation, are influenced by attitudes, values, and beliefs,^26^ including ethical values.

Implementation fidelity refers to the extent to which an intervention is delivered as intended, aligning with the stipulated policy document and its agreed-upon value sets. It's measured in various ways,^27^ including adherence, dose, quality of delivery, participant responsiveness, and program differentiation.^28^ This alignment, however, does not inherently question the ethical soundness of the intervention itself. While some use implementation fidelity and integrity interchangeably,^29^ the authors of this article wish to distinguish between them. Implementation integrity extends beyond mere alignment and adherence; it considers whether the implementation was carried out ethically, fairly, transparently, and without distortion or corruption. It demands an ethical assessment of the entire process, including the exercise of discretion and the impact of the intervention.

A clash of values or value misalignment can occur at various levels—for example, between the normative values of a policy and the personal values of the policy implementer, or between cultural and ethical values. Since values can be conflicting, it's crucial for public health practitioners and policymakers to develop and promote core value sets that can motivate both implementation fidelity and integrity for better public health practice. This integration should begin in both pre-service and in-service training.

An example of a lack of implementation integrity is the inequitable response to public health emergencies and the unequal access to interventions, as seen with COVID-19 and malaria. The speed and scale of a response often depend on the affected population, creating a Global North–Global South divide in public health response. When the Global North faces a high disease burden (e.g. COVID-19), the response is rapid; research, development, and deployment that would typically take a decade were completed in a single year.^30^ In contrast, for diseases like malaria that primarily affect low-income populations in Africa, the deployment of promising technologies like gene drives^31^^,^^32^ has not been prioritized despite high mortality rates.^33^ This disparity clearly demonstrates an inequitable response.

Therefore, the comprehensive integration of ethics, values, and law into public health curricula, policy development, practice and decision-making is not merely desirable; it is absolutely critical for navigating the complexities of a multipolar world and achieving sustainable, equitable, and ethically sound public health outcomes for all, including the global South.

## The VEDMAP framework as a tool for implementation fidelity and integrity for public health programming

The VEDMAP (Value- and Evidence-Based Decision Making and Practice) framework of Mfutso Bengo *et al*.^34^ is a tool for the conscious use of values in public health decision-making. Though not detailed here, it makes transparent which values were selected as necessary and relevant for a given public health decision. The framework demonstrates that effective public health decisions require not only appropriate evidence to inform good policy, but also appropriate values to influence professional behavior, practice, and ultimately, ensure successful implementation.

Although ethical values have historically been sidelined or insufficiently emphasized—for example, ethics did not form an explicit building block in the WHO's original health systems framework—there is a growing call to consider ethics in public health policy assessment.^35^ We contend that without the proper integration of ethics, values, and law into public health initiatives, achieving sustainable and equitable outcomes will remain exceedingly difficult.^36^ Fundamentally, values influence individual and collective behavior change, which is the bedrock of successful public health promotion.

While there has been a necessary emphasis on evidence-based decision-making, both evidence and values are equally critical for sound public health policy and practice. The demand and supply of public health evidence themselves^37^ require value sets that genuinely support its generation, dissemination, and utilization. In essence, while robust public health evidence can inform good policy, appropriate values are what truly influence professional behavior and lead to good practice. VEDMAP makes the utilization of values a conscious process, creating a visible footprint of decision-making. This promotes transparency and accountability and allows for the review of the discretionary exercise of administrative and executive powers.

## Conclusion

Public health policy and practice are increasingly shaped by geopolitical shifts and a consequent clash of values. The transition from a unipolar to a multipolar world has disrupted the dominance of Western standards in public health governance. While globalization initially fostered a standardization of ethical and social values, contemporary movements now emphasize local identity, cultural divergence, and diversified governance. The future of global public health therefore requires navigating these competing value systems to achieve a balanced and sustainable model for ethical and equitable policy-making and implementation. Given the demonstrated inequitable public health responses, the formal inclusion of ethics, values, and law in public health policy and practice is crucial. In order to achieve value convergency in public health there is a pressing need to develop appropriate, core public health values, principles, and codes of ethical conduct^38^ to guide implementation fidelity and integrity.

## Notes

**Joseph Mfutso Bengo,** Professor for Bioethics and Behaviour Studies, Director of Health Economics and Policy Unit

**Eva Mfutso Bengo,** Volljuristin, Acting Head of Department of Economics and Law
